# Pix Proteins and the Evolution of Centrioles

**DOI:** 10.1371/journal.pone.0003778

**Published:** 2008-11-20

**Authors:** Hugh R. Woodland, Andrew M. Fry

**Affiliations:** 1 Department of Biological Sciences, University of Warwick, Coventry, United Kingdom; 2 Department of Biochemistry, University of Leicester, Leicester, United Kingdom; Institute for Research in Biomedicine, Spain

## Abstract

We have made a wide phylogenetic survey of Pix proteins, which are constituents of vertebrate centrioles in most eukaryotes. We have also surveyed the presence and structure of flagella or cilia and centrioles in these organisms, as far as is possible from published information. We find that Pix proteins are present in a vast range of eukaryotes, but not all. Where centrioles are absent so are Pix proteins. If one considers the maintenance of Pix proteins over evolutionary time scales, our analysis would suggest that their key function is to make cilia and flagella, and the same is true of centrioles. Moreover, this survey raises the possibility that Pix proteins are only maintained to make cilia and flagella that undulate, and even then only when they are constructed by transporting ciliary constituents up the cilium using the intraflagellar transport (IFT) system. We also find that Pix proteins have become generally divergent within Ecdysozoa and between this group and other taxa. This correlates with a simplification of centrioles within Ecdysozoa and a loss or divergence of cilia/flagella. Thus Pix proteins act as a weathervane to indicate changes in centriole function, whose core activity is to make cilia and flagella.

## Introduction

The core of this paper is a phylogenetic analysis of Pix proteins, which are constituents of vertebrate centrioles [1]. A technical problem with establishing the function of these proteins is that they are very stable, thus making them hard to deplete using methods such as RNA interference and the resulting phenotypes are not very informative because, as with some other components of centrioles their malfunctions leads to the activation of cell cycle checkpoints or apoptosis [2], [3]. We have therefore used a phylogenomic approach to identify key aspects of Pix function conserved in evolution. We find that Pix proteins are found across a vast range of eukaryotes, but are absent from some. Our analysis suggests that the key function on evolutionary time scales of Pix, and more importantly centrioles, is to make cilia and flagella. Further, the Pix proteins themselves are only needed to make cilia and flagella that undulate, and even then only when (as is generally the case) they are constructed by transporting ciliary constituents up the cilium using the intraflagella transport (IFT) system. In addition we find evidence of a general divergence of Pix proteins in Ecdysozoa , which correlates with diminished importance of cilia and flagella and a simplification of centrioles.

### Centrioles and Basal Bodies

The most thoroughly studied role of the centriole is to maintain the integrity of the centrosome, the principal microtubule organising centre (MTOC) of animal cells. In this context the centriole has a major function in formation of mitotic and, in many cases, meiotic spindles [4]. A second role is in organising cilia and flagella, where centrioles are also known as basal bodies [5]. In an organism like the unicellular, flagellated, photosynthetic protist *Chlamydomonas*, these two functions are mutually exclusive, so cells are either motile or dividing [6]. This may well represent the situation that existed in very early eukaryotes.

The centrioles are typically present as pairs of orthogonally placed cylinders of microtubules, each composed of 9 sets of triple tubules [9(3)] [7]. Although centrioles can be constructed de novo, they typically arise from pre-existing centrioles by a semi conservative process, so that each centrosome contains a young and old centriole, the daughter and the mother [8], [9], [10]. The 9(3) structure of centrioles is comparable to the typical structure of eukaryotic cilia and flagella, except that these usually have 9 sets of doublets surrounding a central pair of singlet tubules [9(2)+2]. This core microtubular structure is known as the axoneme. However, in recent years it has become apparent that many animal cells have a single cilium without the central pair of tubules [9(2)+0]. These “primary” cilia usually have a sensory function and they are non-motile, except for some of those in the principal signalling centre of early vertebrate embryos, the node or organiser, where they are involved in directing left/right asymmetry [11], [12].

In a conventional, undulatory cilium the 9(3) centriole grades into the 9(2) structure of the ciliary axoneme, which it constructs, explaining the fundamental similarity of the two structures. In contrast, when a centriole is involved in organising and initiating the formation of the microtubules of an interphase cell, or the spindle of a dividing cell, it acts as a scaffold focusing a mass of other proteins, including γ-tubulin ring complexes, which actually perform these roles. This larger organelle is called the centrosome and there is no obvious link between its function and the 9(3) structure of centrioles. The centrosome also contains regulatory proteins concerned with progression through the cell cycle, some of which are associated with the centriole itself [4].

### Pix proteins

Pix proteins were discovered in *Xenopus* oocytes because they interacted with a *Xenopus*-specific protein called Xpat, which is a constituent of germ plasm [1]. Germ plasm is a granular structure localised into the vegetal cortex of the egg and contains dense aggregates of RNPs and mitochondria. It is inherited by a small number of cells in the blastula and directs them to become the germ line. Ectopic Xpat itself can form germ plasm-like structures [13], which made its interaction with Pix interesting. Importantly, Pix proteins turn out to be highly conserved in other vertebrates and beyond.

In cultured cells Pix proteins localise to mitochondria in a microtubule-dependent fashion [1]. This most likely explains why Pix is localised to germ plasm, because it is rich in mitochondria. We also found that Pix is localised to mitochondria in the embryos of a sister group of vertebrates, the ascidians, in particular into the embryonic mitochondria of the yellow crescent, an area of cytoplasm that will form the muscles of the larva (Sardet, Paix and HRW, unpublished observations). Thus, the mitochondrial localisation of Pix is likely to be a general phenomenon, at least in Deuterostomes, the clade containing vertebrates. However, in both mammals and frogs, we found that Pix proteins are also constituents of centrioles. Consistent with this location, injection of Pix antibodies into cultured cells causes abnormalities of cell division [1]. In all vertebrates examined there are two Pix genes, encoding similar proteins called Pix1 and Pix2, which both localise to centrioles. In addition, Pix1 and Pix2 were identified as components of the human centrosome proteome, while Pix1 was identified as a component of the mouse photoreceptor ciliome complex [14], [15]. In the protist *Chlamydomonas* the Pix homologue is Poc1 (see below).

In this paper, we describe the wider conservation of Pix proteins and show that, while the protein is conserved in organisms with undulatory cilia, it is absent wherever these structures are lacking or immotile. Beyond this, we argue that in organisms where motile cilia are lacking, centrioles disappear, and where the motility of cilia or flagella is absent or poor, centrioles diverge from the conventional structure. This is reflected by loss or divergence of Pix proteins. This suggests that the principal conserved function of centrioles is to make undulatory cilia or flagella, and of Pix is to enable centrioles to achieve this function. We then speculate on how the link between centrosomes and spindles might have arisen.

## Analysis

### Taxonomic distribution of Pix proteins

**Figure 1:**
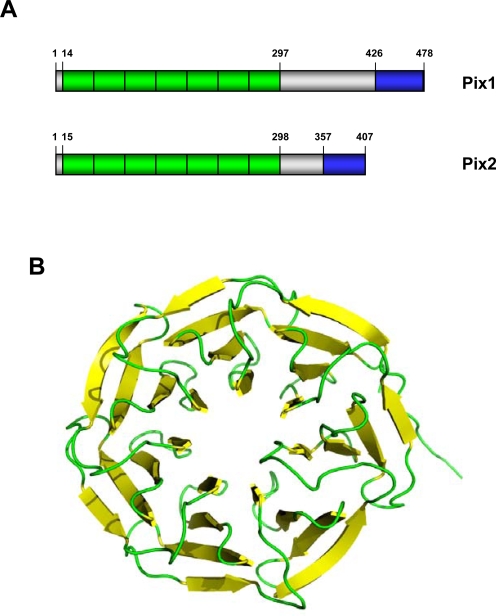
Pix protein organization. A. A schematic diagram of the two human Pix proteins with amino acid numbers indicated. Pix proteins consist of an N-terminal domain containing seven WD40 repeats (green) and a highly conserved C-terminal coiled-coil (blue). B. A model of the Pix protein WD40 repeat domain folded to form a β-propellor. The structural model was built using MODELLER with the structure of the WDR5 protein (pdb-entry: 2GNQ) serving as a template. The figure was generated in pymol.

**Figure 2:**
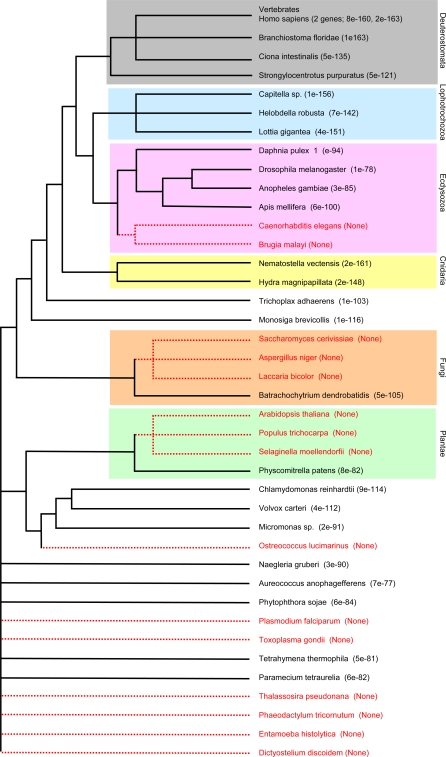
Pix proteins across eukaryotes. A cladogram of a wide range of organisms is drawn according to the current, generally accepted consensus. Pix genes were identified by BLAST search of genomes and identified as hits to the N-terminal 7 WD-40 repeats and the C-terminal coiled-coil region. When these proteins were in turn used to search all genomes their closest vertebrate homologues were Pix proteins. The presence of *Pix* genes in the genome is indicated by black entries and their absence by red. Pix sequences were compared to *Xenopus laevis* Pix2 by pairwise BLAST and the P-value for the match is shown.

**Figure 3:**
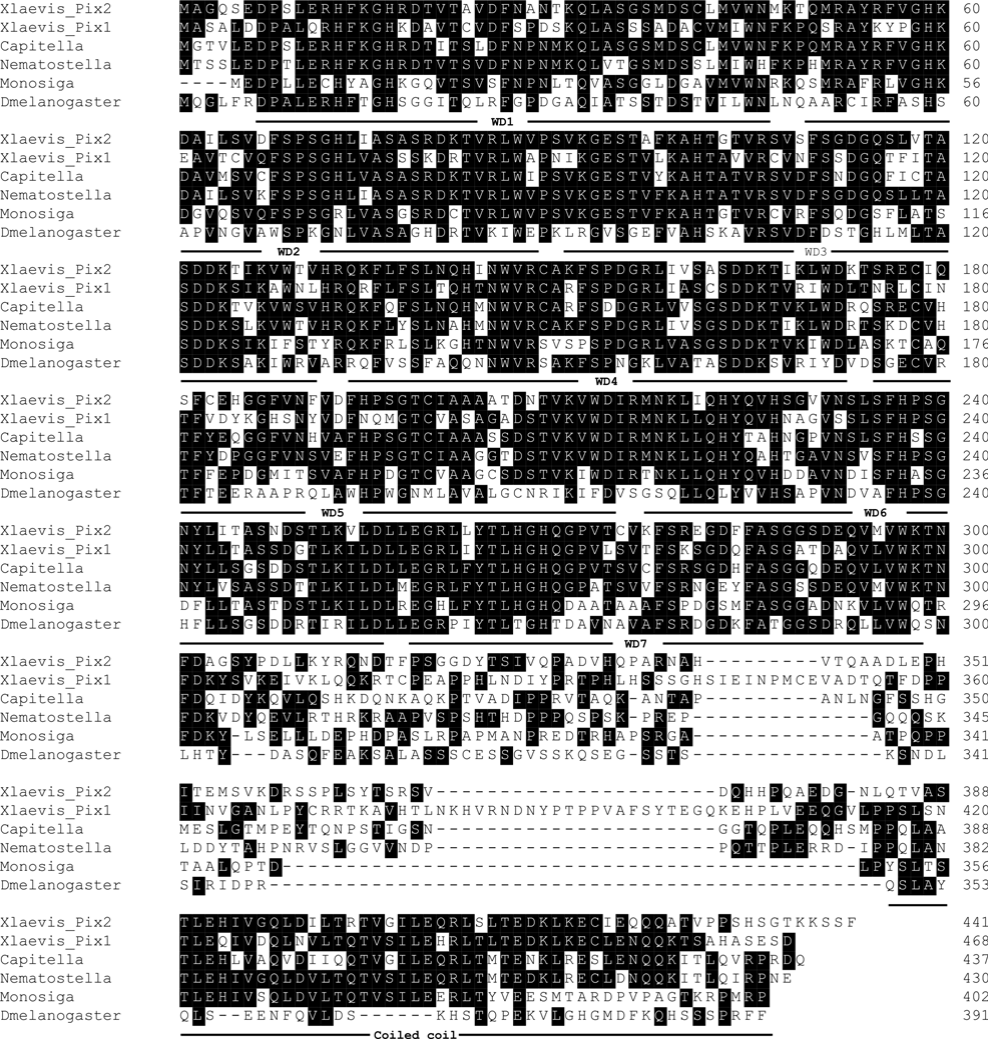
Sequence alignments of Pix proteins from representatives of major animal groups compared by ClustalW. *Nematostella vectensis* (Cnidaria, Anthozoa); *Capitella* sp. (Lophotrochozoa, Annelida); *Drosophila melanobaster* (Ecdysozoa, Arthropoda); *Xenopus laevis*, with two *Pix* genes (Deuterostomata, Vertebrata); *Monosiga brevicollis* (Choanoflagellida). Identical amino acids blocked in black and domains are identified according to the *Nematostella* sequence, using the programs SMART and Coils at EMBL-EBI.

The Pix proteins were first discovered in *Xenopus* and humans and are characterised by two conserved structures: an N-terminal region containing seven WD40 protein repeats and a small but highly conserved coiled-coil region near the C-terminus [1]. Based on homology with other WD40 repeat proteins and modelling studies of the Pix WD40 repeats, it is expected that this domain folds into a β-propellor structure that provides a surface for protein-protein interactions. (Figure 1). However, while there are many proteins with seven WD40 repeats, only one or two per organism can be found with the conserved C-terminal coiled-coil motif. BLAST searches of these against the protein database always show them to have great similarity with the vertebrate Pix proteins (Figures 2, 3; Table S1).

Two Pix proteins were found in all the vertebrate genomes examined, corresponding to Pix1 and Pix2 in humans and *Xenopus*. In other Metazoa there are either one or none. The result of genomic BLAST searches are summarised in Figure 2, which is a cladogram of eukaryotes based on a consensus of molecular evidence, and Figure 3 which shows an alignment of Pix proteins from representatives of major animal groups. Organisms where Pix could not be identified are marked in red. A crude measure of Pix relatedness is indicated by pairwise BLAST P-value scores for the Pix sequence in question compared to *Xenopus* Pix2 (the P value indicates the likelihood that the similarity occurs by chance).

Compared to vertebrates the similarity with the Pix sequences of Cnidaria is remarkable. Cnidaria are basal metazoans diverged from bilaterally symmetrical animals about 600 Mya. Using Clustal W to make comparisons, the sea anenome *Nematostella vectensis* Pix is 59% identical to *Xenopus* Pix1 and 65% to *Xenopus* Pix2, whereas the *Xenopus* proteins are only 55% identical to each other. Multicellular animals evolved from flagellates, specifically the Choanoflagellata, represented by *Monosiga brevicollis*. *Monosiga* Pix is 53% and 51% identical to *Xenopus* Pix1 and Pix2, respectively. If one looks at the other bilaterally symmetrical groups, Pix sequences of the Lophotrochozoa are similar to vertebrates, with P values of about 1e-150. These organisms, including molluscs and annelids, are heavily dependent on undulatory cilia/flagella at various stages of their life cycle.

It is equally instructive to ask in which organisms Pix cannot be identified. In multicellular animals the sole examples identified to date are *C. elegans* and other nematodes. Of course such an absence might be because of incomplete genome coverage, but since it is absent in a range of available nematode genomes, this loss is likely to be real. Nematodes are members of the Ecdysozoa (Figure 2, pink box) and the Pix proteins of non-nematode members of this clade are as diverged from other multicellular animals as they are from those of ciliates, a group of organisms that branched off very early in eukaryote evolution. The significance of this divergence is discussed in detail below.

Amongst plants, angiosperms also lack Pix, but mosses do not. Similarly fungi, including yeasts, lack Pix, except for the primitive, parasitic chytrid fungus *Batrachochytrium dendrobatidis*. Interestingly, higher plants lack centrioles and cilia/flagella, as do most fungi. However, mosses have flagellated zoospores, as does *Batrachochytrium*. This suggests that the presence of Pix proteins correlates with the presence of flagella. A similar argument can be made in protists, where amoebae which lack Pixl also lack flagella. Thus, there is an obvious correlation between the presence of Pix proteins and the possession of cilia/flagella in various groups. There are exceptions however. Pix could not be found in the *Selaginella* genome, but members of this clubmoss group have haploid, flagellated zoospores. This could result from a lack of full genome coverage, so further work is needed to clarify this situation. However, centrioles form in a unique way to generate the flagella in this group of simple land plants, the Lycopodiaceae, as they do in pteridophytes [16], [17], [18], so one could be detecting first steps in the divergence and subsequent loss of flagella in early land plants.

Another apparent exception may not be real. The marine bloom organism *Aureococcus anophagefferens* has Pix, but has not been reported to have flagellated cells in its life history. These organisms have not been intensively studied and it is possible that zoospores have been missed. Other Pelagophyceae have flagellated zoospores (Chrysonephos) or basal bodies (Pelagococcus), supporting the likelihood that a flagellated stage exists in *Aureococcus*. This view is also supported by the presence in the *Aureococcus* genome of a β-tubulin with the C-terminal motif essential to form 9+2 cilia. Organisms without 9+2 cilia lack such tubulin. [19], [20]. The single-celled alga *Chlorella*, which is not known to have a flagellated stage, has a Pix homologue and a β-tubulin that is related to the flagellar type. This might support the idea of an unknown flagellated form of *Chlorella*, but the fact that *Aureococcus* contains a variety of IFT genes but *Chlorella* does not, would make it more likely that, while the former has an undiscovered flagellated stage, *Chlorella* does not and uses Pix in some unusual way.

Other protists support the hypothesis that the presence of Pix homologues correlates with orthodox undulatory cilia/flagella. We have already mentioned the presence of a highly conserved Pix in a choanoflagellate. This is true of photosynthetic flagellates related to multicellular plants (*Chlamydomonas*, *Volvox*, *Micromonas*), indeed in *Chlamydomonas* the Pix homologue (Poc1) has been identified in the flagellar proteome [21]. Pix is also present in other flagellates and ciliates, but is absent from *Entamoeba* and *Dictyostelium*, all of which lack cilia/flagella. In *Tetrahymena* a Pix (Poc1) homologue was identified in the basal body proteome. EM immunocytochemistry shows it to be localised to the basal end, or cartwheel of mature centrioles and to the amorphous assembly disc of newly forming centrioles [22]. This is different from Pix localisation in vertebrate centrioles, which is preferentially to the distal end [1]. This difference may be related to the absence of this cartwheel centriolar precursor in animals.

However, there are several protists which have flagella, but apparently lack Pix. These cases turn out to be provocative, because they make their flagella in an unusual way. Apicomplexans, such as the malarian parasite *Plasmodium*, have flagella that seem to be simpler than those of other eukaryotes, and their genomes lack IFT genes to transport components into the flagellum. In this case the axonemes are constructed within the main cell body [23], which is similar to the process by which the sperm axoneme is made in *Drosophila*, see below (review [24]). A second example is the centric diatom *Thalassiosira*, which is deficient in Pix and IFT genes and has flagella with a 9(2)+0 axoneme [25].

Finally, there is direct evidence that the ciliary/centriolar function of Pix proteins is highly conserved in eukaryotes, since Pix proteins have been identified in the basal body proteome of *Chlamydomonas* [26] and *Tetrahymena* [22]. Overall the presence of Pix, the β-tubulin motif, and undulatory cilia/flagella is correlated (Table S1), but there are several apparent exceptions which deserve further investigation.

### Pix in Ecdysozoa

**Figure 4:**
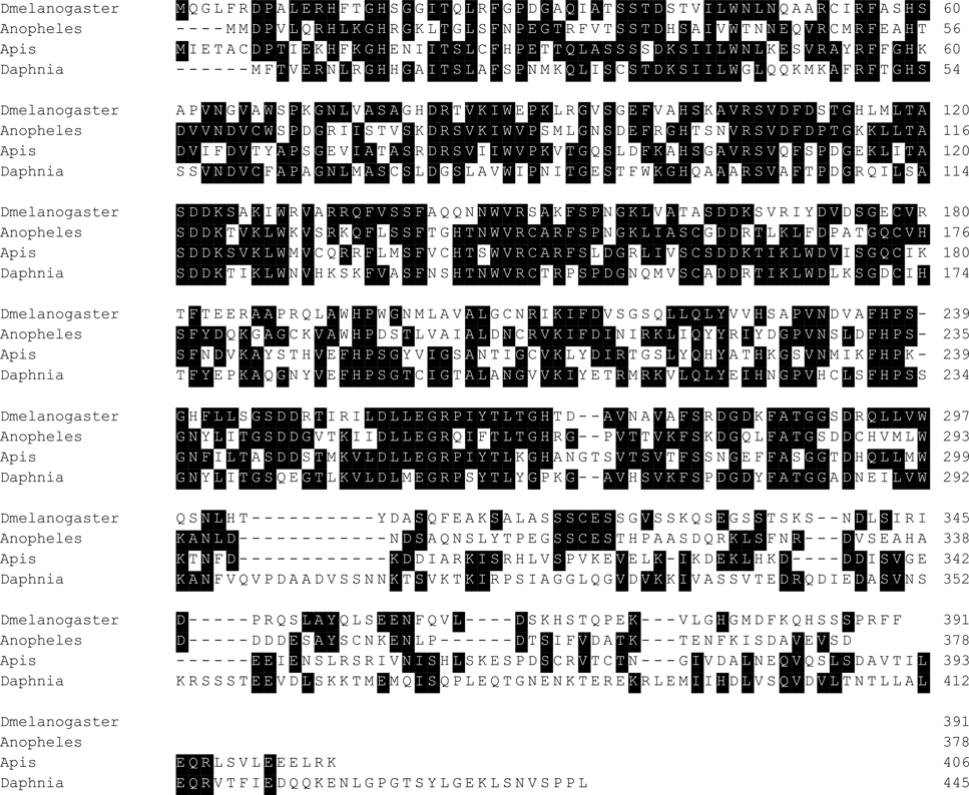
Comparison of Pix proteins in Ecdysozoa. ClustalW was used to compare the Pix proteins of *Drosophila melanogaster (Insecta, Diptera)*, *Anopheles gambiae* (Insecta, Diptera) *Apis melifera* (Insecta, Hymenoptera), *Daphnia pulex* (Crustacea, Cladocera). Details as in Figure 3.

The absence of Pix in nematodes and its divergence in other Ecdysozoa has already been mentioned. One characteristic of Ecdysozoa is the absence of undulatory cilia, except in the sperm of some groups [27]. Ecdysozoa are characterised by an inert, moulting cuticle, which precludes the presence of ectodermal locomotory cilia [28]. Within the Ecdysozoa *Drosophila* has the most divergent Pix sequence identified in any Metazoan (1e-78) and several other insects are only a little less diverged (Figures 2, 4; Table S1). The crustacean *Daphnia* falls into the middle of this range. While, compared with other animals, there is some sequence conservation in the C-terminal region, in Diptera the Coils program predicts only a low probability that it will form a coiled-coil. On the other hand the probability is very high in *Daphnia* (Crustacea) and Apis (Hymenoptera), even though the sequence is quite diverged. This suggests that this region of the protein may have lost its conserved function in dipteran flies, and that selection is relaxed in other Ecdysozoans.

What is special about Ecdysozoa? Neither nematodes nor most crustaceans, including *Daphnia*, have flagellated sperm, although all have sensory primary cilia (9+0). Thus these organisms totally lack locomotory cilia. In insects the occurrence of primary cilia is restricted to Type I mechanoreceptors, so their dependence on sensory cilia is far less than in vertebrates [29]. In the insect *Drosophila melanogaster* sperm are flagellated, yet they are truly remarkable because the sperm tail is as long as the male. In *Drosophila bifurca* they are forty times longer than the male, that is 58 mm [30]! It is hard to imagine that such sperm ever undulate in any organised way; rather the great length is likely to be an adaptation to sperm competition in a species in which females eject sperm before mating again with a new partner. Bees have more normal sperm length, but the axonemes are unusual in having 9+9+0 structure (Zama et al., 2005). Bee Pix is a little more like vertebrate Pix than that of *Drosophila*. This suggests that the axonemes of the flagella of insects may be different from those in other animals at a molecular level. Interestingly, the way in which sperm flagella are made in *Drosophila* is unusual and is more like that in *Plasmodium* and diatoms described above, that is the axoneme is constructed in the main cytosol [31] without the function of IFT proteins [32], [33]. On the other hand the sensory cilia of *Drosophila* do require IFT proteins.

Thus, undulatory cilia/flagella have become simplified and less employed in the evolution of Ecdysozoa and they are absent in nematodes and most crustaceans. However, all members of the clade possess 9+0 primary, sensory cilia, although these are far less used than in animals like vertebrates. This suggests the generalisation that Pix proteins are essential to form typical motile cilia/flagella, but not necessarily the non-locomotory, primary kind. Since ecdysozoan Pix proteins are more diverged than in any members of the clade, including Choanoflagellates and multicellular animals, it suggests that the requirements of a Pix to make primary cilia are less demanding than in making normal undulatory cilia. In addition, the phylogenomics suggest that, at least in the long term, Pix proteins are only essential to make cilia/flagella via an IFT-dependent mechanism. Since Pix proteins are localised within the lumen of the centriole, rather than in cilia/flagella [1], it is reasonable to suggest that Pix proteins are needed to make the sort of centriole capable of making cilia/flagella via IFT transport processes. Of course other proteins are also needed to do this, including β-tubulin with a specific tubulin motif (EGEF followed by 3 acidic residues; Table S1) [19], [34]. These suggestions raise further questions about the core function of centrioles.

### Centrioles and Pix in diverse organisms

Higher land plants and fungi (other than *Batrachochytrium*) lack cilia/flagella, centrioles and Pix. Centriolar structure in *Drosophila* is interesting because only in sperm development are long centrioles with a 9(3) structure found and, as already described, the sperm flagellum is made in an atypical, IFT-independent manner. In other tissues centrioles may be 9(2) or even 9(1) [35]. This suggests that the centriole is becoming less typically organised as cilia/flagella become less important. It is significant that the later development of *Drosophila* is possible without centrioles [29], [36], [37]. This may be enabled by a generally reduced functionality of centrioles in this clade. While comparable experiments have not been performed in other animals, mammalian cultured cells divide abnormally in the absence of centrioles [38], suggesting that mammalian development would be impossible without centrioles. In fact, in *Drosophila* that lack centrioles as a result of DSas-4 deficiency, lethality does ensue when combined with loss of the mitotic checkpoint protein, Mad2, even though Mad2 loss alone is not inviable (J. Raff, personal communication). Further, larval brains often develop malignant neoplasms in Dsas-4 deficient flies [39], suggesting that a role for centrioles in cell division remains important.

*C. elegans* lacks undulatory flagella, having amoeboid sperm, but has primary 9+0 sensory cilia. Its centrioles are of a single tubule, 9(1) kind [40]. Again this is consistent with the evolutionary loss of conventional cilia leading to a simplification of centrioles and a concomitant loss of Pix proteins. This simplification extends to the loss of other proteins from both *C. elegans* and *Drosophila*, namely δ- and ε-tubulin [41], [42].

Together, these observations support a hypothesis that the core function of centrioles across eukaryotic phyla is to construct either the conventional motile 9(2)+2 secondary cilia or 9(2)+0 primary cilia. Pix is essential only for the former, and then only when they are made via an IFT-dependent mechanism.

## Discussion

### What are conventional centrioles for?

As explained, the presence of Pix, undulatory cilia/flagella and conventional centrioles correlate across the eukaryotic phyla. Thus, when only primary cilia are present centrioles are simplified (*C. elegans*). In *Drosophila* there are primary cilia and the sperm are flagellated, but their undulatory movement cannot be normal. Here Pix is divergent and in somatic tissues at least the centrioles are simplified. These organisms have primary, 9+0 cilia, for which a reduced centriole is sufficient. In advanced land plants and most fungi, without even primary cilia, the loss of all cilia has led to the loss of centrioles. Broadly speaking these correlations are supported across protists. Apparent exceptions like diatoms and apicomplexans have flagella, but no IFT genes and intra-cytosol manufacture of the axoneme.

The main conclusion of these observations is that the core conserved function of centrioles is to construct flagella/cilia, but that if these are not of the undulatory 9(2)+2 kind a less sophisticated centriole will do (see also discussion by Marshall [5]). Without this function selection does not maintain centrioles at all, at least on evolutionary time scales. This makes sense because the structure of the centriole corresponds to that of the axoneme, indeed it blends into it from the basal body. On the other hand centriolar structure has no relationship to the microtubules nucleated by MTOCs. In this role centrioles merely act as a platform for aggregating MT nucleating proteins. Typically, in mammalian cells the centrioles organise a bipolar division spindle, prevent multipolar spindles forming, and control aspects of progression through the cell cycle. It is essential that these processes are precisely regulated or chromosomal missegregation may occur.

### How did centrioles become associated with the division spindle?

Flagella clearly evolved in very early eukaryotic cells [43]. Ciliates are an early diverged offshoot, but typically the early protists would have had a single flagellum or a pair, in each case arising from a single basal body, or centriole. Although centrioles can arise de novo it would clearly be advantageous for each mitotic daughter cell to be able rapidly to assemble new flagella using a basal body, and hence to swim. Thus, there would have been selection for a robust mechanism to supply each daughter with a single centriole. On the one hand there would have to be robust control of centriolar replication, tightly linked to the cell cycle. On the other hand association of the centrioles with the spindle poles would have ensured that each centriole would arrive in a different daughter cell.

One can envisage that there would have been progressive integration of the centrioles into other aspects of cell division. This might be compared to situations where parasitism evolves towards symbiosis. Initially, the centriole has “parasitized” the spindle, then the two have become mutually dependent. The tight linkage of centriolar replication to the cell cycle would have led to the centriole becoming a platform for molecules regulating the cell cycle and controlling the number of spindle poles, rather than simply using them for localisation. Such a role would be consistent with the observation that *Chlamydomonas* without centrioles can still divide, albeit with abnormal cell division and slow growth caused by disorganized mitotic spindles and cytoplasmic microtubules [44]. Of course plants and fungi tell us that while this role may be advantageous, without the role of centrioles in constructing cilia/flagella their existence is unsupportable in the long term. It is noteworthy that these organisms have rigid cell walls, which may have enabled control of cell division by other means. Furthermore, while centrioles are largely dispensible in the later development of *Drosophila*, they are essential for the early divisions, when the embryos are syncytial [36]. This reduced dependence on centrioles may be aided by the fact that the requirement for centrioles is relaxed in ecdysozoans, but apparently centrioles are still absolutely necessary when there is not even a cell membrane for astral microtubule attachment.

To support these proposals further work is clearly needed. Exceptional situations, like *Selaginella*, should be clarified. *Drosophila* provides an interesting test, since centrioles have different degrees of complexity in different tissues. If Pix is knocked out would it affect only sperm, or sensory neurones, or other tissues as well? And are other centriolar proteins divergent or absent in a way that correlates with Pix? In *Chlamydomonas* centrioles alternate between essential spindle roles and constructing flagella. So what would disruption of Pix do, indeed what is the precise function of Pix in any organism? While there are many experimental lines that need investigation, the argument for a core role of centrioles in making cilia/flagella, while largely non-experimental, is still a very strong one. Moreover the phylogenetic survey of centrioles certainly throws up interesting trends, particularly that of simplification of centrioles in the Ecdysozoa.

## Supporting Information

Table S1 Survey across eukaryotes of centrioles, cilia, flagellum-specific β-tubulin and Pix homologues. Deuterostomia (white), Ecdysozoa (blue), Lophotrochozoa (grey), Cnidaria (yellow), Fungi (pink), plants and protistan sister groups (green), other protists (purple). Column 3, taxonomic groups are from the NCBI taxonomic database. The β-tubulin cilia/flagellum C-terminal domain was sought in genomes using BLAST with the Drosophila sequence (EGEFDED; the human sequence is EGEFDEE and consensus is EGEF+3 acidic residues[19]). The absence of this protein from the puffer fish genome is unlikely to be real. Column 8, Pix homologues were sought in genomes by BLAST with the Xenopus Pix2 sequence. The diagnostic feature of the Pix proteins was taken to be seven WD40 repeats plus homology in a coiled coil region in the C-terminus. The proteins were re-BLASTed again and their closest relatives were known Pix genes. The presence of the C-terminal coiled-coil region was confirmed using the program COILS (http://www.ch.embnet.org/software/COILS_form.html). The number of Pix homologues in genomes is shown in brackets. The similarity of Pix homologues is represented by a BLAST similarity score with the Xenopus Pix2 sequence.
(0.03 MB XLS)
